# Supplementary material for: The ECM and tissue architecture are major determinants of early invasion mediated by E-cadherin dysfunction
Source: Commun Biol. 2023 Nov 8;6:1132. doi: 10.1038/s42003-023-05482-x (PMC10632478; doi:10.1038/s42003-023-05482-x)
Supplement: Supplementary file 2 — Supplementary Information File [file 42003_2023_5482_MOESM2_ESM.pdf]

Supplementary Information for: "In vitro and mathematical strategies uncover the extracellular matrix and tissue architecture as major determinants in early steps of invasion mediated by E-cadherin dysfunction"

Soraia Melo, Pilar Guerrero, Maurício Moreira Soares, José Rafael Bordin, Fátima Carneiro, Patrícia Carneiro, Maria Beatriz Dias, João Carvalho, Joana Figueiredo, Raquel Seruca, Rui Travasso

Development of a Dissipative Particle Dynamics (DPD)-based model

Distinct mathematical models may provide different perspectives on a physical system. Herein, aside the phase-field and the vertex models, we have proposed a minimal Dissipative Particle Dynamics (DPD) model inspired on a membrane model [1]. This approach enables to run a large number of simulations, yielding qualitative statistical information with regards to the dependence of the system dynamics on the adhesive features of individual cells.

For this purpose, each cell was modelled as a dumbbell particle, with two monomers or beads, linked by a rigid constraint at a distance equal to the monomer diameter $\sigma = 1.0$ (for simplicity, all quantities are presented in the standard Lennard Jones (LJ) reduced units [2]). The beads constituting wild-type and mutant cells will be referred by w-beads and m-beads, respectively. The linkage between beads was kept rigid through the SHAKE algorithm [3]. To model the extracellular matrix (ECM), a crowded media composed of non-overlapping spherical obstacles with diameter σ was implemented, corresponding to the third species in the system, namely the ecm-bead.

The influence of tissue 3D structure in cell fate was investigated in flat and cylindrical models (Fig. S1A-B). The w-beads were first inserted in the desired geometry, and the ecm-beads were then randomly placed below the flat tissue or surrounding the cylindrical tissue. Then, one random wild-type cell was chosen to mutate, thus becoming constituted by two m-beads, and losing its adhesion to its neighbours cells. All interactions were generated by the Lennard-Jones potential with cosine tail [4], which allowed to turn on and off the short range attraction between beads, thus reproducing the establishment and disassembling of cell-cell contacts. Specifically, the LJ interaction potential is described as follows:

$$U_{\mathrm{LJ}}(r_{ij}) = 4\varepsilon_{ij}\left[\left(\frac{\sigma}{r_{ij}}\right)^{12} - \left(\frac{\sigma}{r_{ij}}\right)^{6}\right] , \qquad \text{for} \quad r_{ij} \leq r_{min} = 2^{\frac{1}{6}}\sigma , \tag{1}$$

where $r_{ij} = |\vec{r}_i - \vec{r}_j|$ is the distance between two particles i and j. The distance $r_{min} = 2^{1/6}\sigma$ corresponds to the minimum in the LJ potential [2]. The adhesion between two beads is included through a cosine tail. From r_{min} up to a cutoff distance $r_{cut} = r_{min} + \omega$ the interaction is given by

$$U_{\mathrm{cosine}}(r_{ij}) = \varepsilon_{ij} \cos^2\left[\frac{\pi}{2\omega}(r_{ij} - r_{min})\right] . \tag{2}$$

For mutant beads, the cosine tail is set to zero. Then, m-beads interact with w-beads by the Weeks-Chandler-Andersen (WCA) potential [5], equal to the LJ potential cut at its minimum, Eq. (1), and shifted by $\varepsilon_{m-w} = 1.0$. The interaction between wild-type beads and between w-beads and ecm-beads are given by the LJ+cosine tail potential, with $\varepsilon_{w-w} = \varepsilon_{w-ecm} = 1.0$. To evaluate the effect of increased adhesion between mutant cells and the ECM, we vary the strength of the respective interaction using the LJ+cosine tail potential with $\varepsilon_{m-ecm} = 1.0$, 1.5 and 2.0. These values correspond to the ratio between mutant cell-ECM adhesion and wild-type cell-ECM adhesion equal to 1.0, 1.5 and 2.0, respectively. The case of no adhesion corresponds to a purely hard-core interaction between the mutant cell and ECM beads, given by the WCA potential. The potentials are shown, in reduced units, in Fig. S1C and the correspondent forces are shown in the inset.

Our DPD simulations were performed with a time step of $\delta t = 0.01$ and $\gamma = 1.0$ at reduced temperature $T = 1.0$ using the ESPResSo package [6]. Aside from the effect of the tissue geometry and cell-ECM adhesion, we have also explored the basal extrusion dynamics under distinct matrix densities Φ_M.

To characterise the basal extrusion process, we follow the z-component of the position in the flat case, and the distance $r = \sqrt{x^2 + y^2}$ for the cylindrical tissue. In the flat tissue case, the mutant cell is initially placed at $z = 0.0$ in the rectangular box, and its extrusive potential can be obtained by evaluating the cell's displacement in the

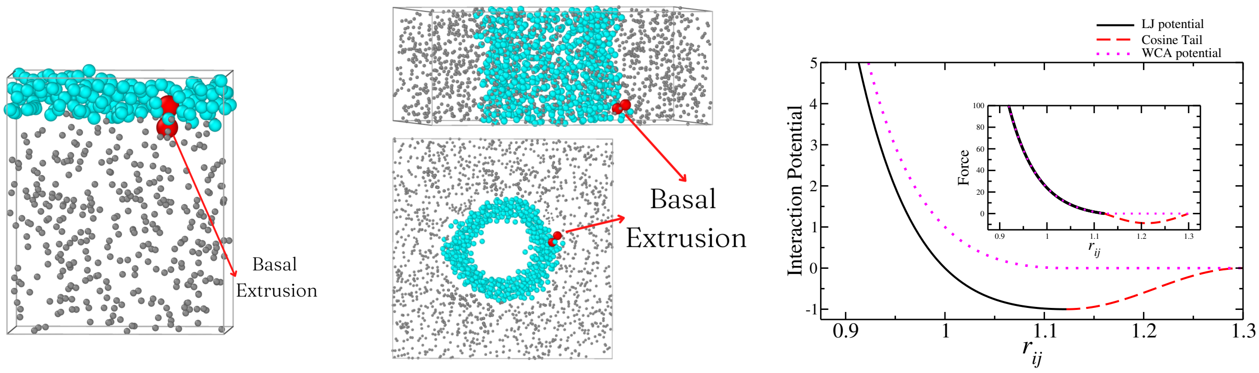

A **B** **C**

Figure S1 . Snapshot showing a flat, A), and a cylindrical, B), tissue of wild-type cells (cyan dumbbells), one mutant cell (red dumbbell) and the crowding media that mimics the ECM (grey spheres). For better visualisation, cyan and grey spheres are displayed with half of their diameter in the simulation. C) Interaction potential between two beads. It consists of a purely repulsive LJ potential up to $r_{ij} = r_{min} = 2^{1/6}$ and an attractive tail from $r_{min} < r_{ij} \leq r_{cut} = 1.3$. Inset: force between a pair of beads. Here $\varepsilon_{ij} = 1.0$.

z-direction, $\Delta z = z(t) - z(0)$. If $\Delta z > 1.0$ the event is considered a basal extrusion, otherwise, an apical extrusion occurs when $\Delta z < -1.0$. In the case of cylindrical tissues, the initial radial position of the mutant cell's center of mass is $r = R$, where R is the cylinder radius. If $\Delta r = r - R > 1.0$, the cell is basally extruded, while, otherwise, an apical extrusion occurs for $\Delta r = r - R < -1.0$.

We have then obtained several cell trajectories, as the ones plotted in Fig. S2A, and evaluated the average velocity of the cell movement. The velocity was calculated by $\langle v_{BE} \rangle = \langle \Delta z \rangle / t$ in the flat tissue, with t being the total simulation time, and by $\langle v_{BE} \rangle = \langle \Delta r \rangle / t$ in the cylindrical geometry, where the average is done over the (at least 50) independent simulations where the cell invades the ECM, out of 100 simulations. In Fig. S2B we plot the mean basal extrusion velocity as function of the tissue curvature, i.e. the inverse of the tissue radius. A flat tissue corresponds to a curvature $1/R_{flat} \rightarrow 0.0$.

This data indicates that basal extrusion velocity is higher in the presence of stronger adhesion to ECM, both in flat and cylindrical geometries, corroborating the results obtained in the phase-field simulations (Figure 4 of the main text). Furthermore, extrusion velocity is predicted to increase with tissue curvature (independently of mutant cell-ECM adhesion), as observed in the phase-field and vertex model simulations (Figures 5 and 6 of the main text).

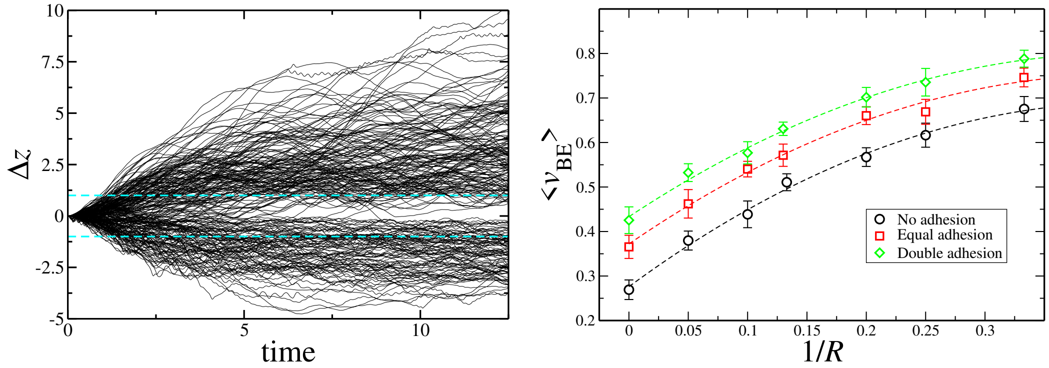

A **B**

Figure S2 . A) Extrusion distance of m-cell as function of time for simulations of flat tissues with distinct ECM densities and distinct cell-ECM adhesion. B) Mean velocity in the radial direction as function of the inverse of the membrane radius for different mutant cell-ECM adhesion (interaction between m-beads and ecm-beads given by the WCA potential and given by the LJ+cosine tail potential, with $\varepsilon_{m-ecm} = 1.0$, and 2.0).

Similar conclusions are drawn by observing in the model the probability for basal extrusion, $P(\Phi_M)$, of the mutant cell as function of the ECM matrix density, Φ_M. In addition to adhesive features and tissue architecture, we found that ECM density may also impact cell extrusive abilities. In fact, we have verified that the probability of basal extrusion is in general inversely correlated with matrix density (with the exception of the region of low density when there is a high mutant cell-ECM adhesion), possibly due to spatial constrictions and limited space for cell movement (Fig. S3). By observing the probability of basal extrusion, and in agreement with the previous observations of the cell velocity and with the phase field model, we observe that a higher adhesion between cell and ECM plays a crucial role in the increase of basal extrusion. Moreover, in the large mutant cell–ECM adhesion scenario, we observe a small increase in the extrusion probability as Φ_M increases in the low density regime. In this regime, though we are decreasing the available space for cell migration, higher adhesion can lead to an increase in the diffusion of the m-beads in a sticky crowded media [7]. Obviously, as we further increase the crowding, the probability of basal extrusion decays. These results are shown in g. S3A for the flat tissue and in Fig. S3B for the cylindrical case, with $R = 5.0$. These results further corroborate that tissue curvature favours basal extrusion. The probability for basal extrusion is higher in higher tissue curvature (Fig. S3B) for similar mutant cell–ECM adhesion and ECM density. In particular, at zero ECM density, we observe a 50% basal extrusion probability (Fig. S3A) for the mutant cell (i.e. neither the apical nor the basal direction is favoured), while the asymmetric pressure that the mutant cell undergoes in the curved geometry, pushes the cell in the basal direction (Fig. S3B).

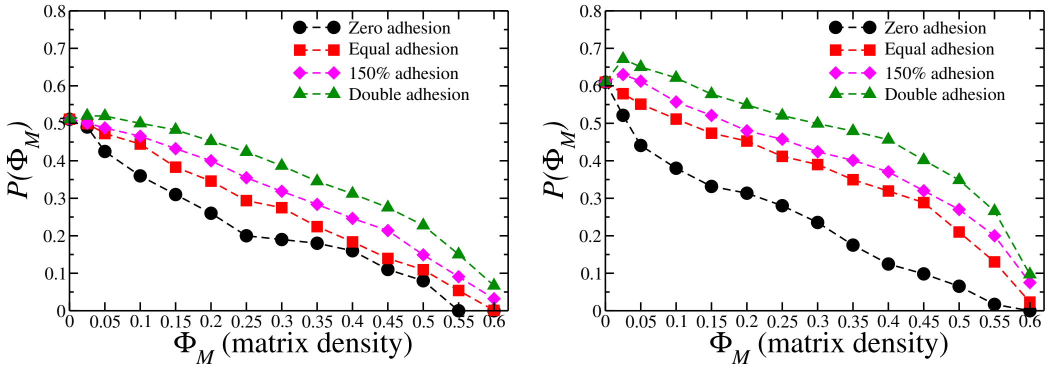

A **B**

Figure S3 . Basal extrusion probability $P(\Phi_M)$ as function of the ECM density Φ_M for the planar A) and cylindrical B) cases with distinct mutant cell–ECM adhesion (interaction between m-beads and ecm-beads given by the WCA potential and given by the LJ+cosine tail potential, with $\varepsilon_{m-ecm} = 1.0$, 1.5 and 2.0).

References

1. Reynwar, B. J., Illya, G., Harmandaris, V. A., Müller, M. M., Kremer, K., and Deserno, M. (2007) Aggregation and vesiculation of membrane proteins by curvature-mediated interactions. *Nature*, **447**, 461–464.

2. Allen, M. and Tildesley, D. (2017) *Computer Simulation of Liquids: Second Edition*. OUP Oxford.

3. Ryckaert, J.-P., Ciccotti, G., and Berendsen, H. J. (1977) Numerical integration of the cartesian equations of motion of a system with constraints: molecular dynamics of n-alkanes. *Journal of Computational Physics*, **23**, 327–341.

4. Soddemann, T., Dünweg, B., and Kremer, K. (2001) A generic computer model for amphiphilic systems. *Eur. Phys. J. E*, **6**, 409.

5. Weeks, J. D., Chandler, D., and Andersen, H. C. (1971) Role of repulsive forces in determining the equilibrium structure of simple liquids. *The Journal of Chemical Physics*, **54**, 5237–5247.

6. Weik, F., Weeber, R., Szuttor, K., Breitsprecher, K., de Graaf, J., Kuron, M., Landsgesell, J., Menke, H., Sean, D., and Holm, C. (2019) Espresso 4.0 – an extensible software package for simulating soft matter systems. *The European Physical Journal Special Topics.*, **227**, 1789–1816.

7. Nogueira, T. P. O., Frota, H. O., Piazza, F., and Bordin, J. R. (2020) Tracer diffusion in crowded solutions of sticky polymers. *Phys. Rev. E*, **102**, 032618.
