# Supplementary material for: The ECM and tissue architecture are major determinants of early invasion mediated by E-cadherin dysfunction
Source: Commun Biol. 2023 Nov 8;6:1132. doi: 10.1038/s42003-023-05482-x (PMC10632478; doi:10.1038/s42003-023-05482-x)
Supplement: Supplementary file 3 — Description of Additional Supplementary Files [file 42003_2023_5482_MOESM3_ESM.pdf]

Description of Additional Supplementary Files

File name: Supplementary Data 1
Description: Experimental data presented in Figure 2; extrusive capacity of E-cadherin mutant cells
